# Clinical characteristics and outcomes of pediatric oncology patients with aggressive biology enrolled in phase I clinical trials designed for adults: The university of Texas MD Anderson cancer center experience

**DOI:** 10.18632/oncoscience.68

**Published:** 2014-07-27

**Authors:** Fernando F. Corrales-Medina, Cynthia Herzog, Kenneth Hess, Daniela Egas-Bejar, David S. Hong, Gerald Falchook, Pete Anderson, Cesar Nunez, Winston W. Huh, Aung Naing, Apostolia M. Tsimberidou, Jennifer Wheler, Sarina Piha Paul, Filip Janku, Eugenie S. Kleinerman, Razelle Kurzrock, Vivek Subbiah

**Affiliations:** ^1^ Children's Cancer Hospital, Division of Pediatrics, The University of Texas MD Anderson Cancer Center, Houston, Texas, USA; ^2^ Department of Biostatistics, The University of Texas MD Anderson Cancer Center, Houston, Texas, USA; ^3^ Department of Investigational Cancer Therapeutics (Phase I Clinical Trials Program), Division of Cancer Medicine, The University of Texas MD Anderson Cancer Center, Houston, Texas, USA; ^4^ Pediatric Hematology/Oncology/BMT, Levine Children's Hospital/Levine Cancer Institute, Charlotte, North Carolina, USA; ^5^ Center for Personalized Cancer Therapy and Division of Hematology & Oncology, University of California San Diego - Moores Cancer Center, La Jolla, California, USA

**Keywords:** phase I trials, children, prognostic scores, targeted therapy

## Abstract

**Background:**

Children (patients ≤ 18 years of age) are not usually included on pharmaceutical industry sponsored Phase I trials.

**Methods:**

We reviewed the medical records of 40 patients ≤ 18 years treated in ≥ 1 phase I trial at MD Anderson.

**Results:**

The median OS was 8.5 months (95% CI, 5.5-13.2 months). In the multivariate analysis, age ≥15 only predicted increased OS (P = 0.0065), and >3 prior therapies (P = 0.053) predicted decreased OS. The median PFS was 2.8 months (95% CI, 2.3-4.1 months). In the multivariate analysis, independent factors that predicted increased PFS were age ≥15 years (P < 0.001) and prior radiation therapy (P = 0.049); performance status >1 (P < 0.001) and >3 prior therapies (P = 0.002) predicted decreased PFS. RMH score ≥ 2 and MDACC score ≥ 3 were associated with decreased median OS (P = 0.029 and P = 0.031 respectively).

**Conclusions:**

It is feasible to conduct phase I studies in pediatric patients based on adult protocols. In the era of targeted therapy more trials should allow pediatric patients earlier in the drug development especially if deemed safe in adults in early phase trials.

**Translational Relevance:**

Most pharmaceutical industry sponsored trials exclude patients less than 18 years in phase I clinical trials. Even in the era of targeted therapy pediatric patients usually have to wait for most phases of trials to be completed in adults before being allowed to enroll in clinical trials of new therapies, even in the advanced metastatic and relapsed setting. Some investigator initiated phase 1 trials of combinations of US FDA approved agents allow patients less than 18 years. We report the preliminary analyses of the outcomes of pediatric patients enrolled in phase I studies initially designed for adults, but allowing for enrollment of patients under 18.

## INTRODUCTION

Frontline and salvage cytotoxic chemotherapy regimens for pediatric cancers have become more complex and intensive in an effort to improve long-term cure rates. [1, 2] The intensification of treatment regimens has been facilitated by parallel improvements in supportive care, growth factor support, and intensive patient monitoring. [2] However, these more intensive frontline regimens may render patients more intolerant of subsequent treatments, including molecularly targeted therapies. (1) Moreover, the intensive regimens may confer aggressiveness to the biology of the disease, making it more refractory to any form of therapy, even if the tumor harbors an actionable aberration.[3] Thus, due to the more aggressive frontline treatments in pediatric cancer therapeutics, new approaches to the clinical development of new agents for the treatment of childhood cancers are needed.[2-11]

Phase I trials play a key role in the early evaluation of novel targeted therapies for patients with advanced cancer.[2, 4, 9, 12]. One of the main challenges of Phase I trials is to select patients who are most likely to benefit from investigational treatments; patient selection is increasingly being facilitated by the identification of molecular markers.[13] Although phase I trials have generally been proven safe, an overall assessment of potential trial participants' predicted survival may further help in the process of selecting patients for a trial.[14, 15] Prior analyses of pediatric phase I trials have focused on the development of standardized recommendations for the trial design, response rates, and observed toxic effects.[16] However, very few published reports have examined the clinical characteristics of pediatric patients at the time they start an investigational drug regimen and how these factors may impact clinical outcomes.[2, 16, 17]

To date, two validated prognostic scores have been shown to help predict survival rates in adults: the Royal Marsden Hospital (RMH) score[14] and the MD Anderson Cancer Center (MDACC) score(11). The RMH score is based on 3 variables associated with poor survival: elevated lactate dehydrogenase (LDH), greater than the upper limit of normal (>618 IU/L), low albumin (<3.5 g/dL), and more than 2 sites of metastasis. The RMH investigators demonstrated that patients with an RMH score of 0-1 had significantly longer overall survival durations compared with patients who had an RMH of 2-3.[14] The MDACC score is based on 5 variables associated with poor survival: elevated LDH (>618 IU/L), low albumin (<3.5 g/dL), more than 2 sites of metastasis, Eastern Cooperative Oncology Group (ECOG) performance status ≥1, and the presence of a gastrointestinal tumor.[13] These scales have not yet been validated in children.

Several investigator-initiated studies in the adult phase I program at MD Anderson have allowed enrollment of children and adolescents.[17] Knowledge of the characteristics and outcomes of children enrolled in pediatric phase I trials designed for adults may be beneficial for future phase I trials, contributing to a better understanding of the risks and benefits for pediatric patients considering enrollment in adult focused phase I trials. The purpose of this study was to determine the relationship between pre-enrollment clinical characteristics and survival outcomes of pediatric patients enrolled in adult-based phase I trials at the Department of Investigational Cancer Therapeutics at MD Anderson Cancer Center. We also sought to correlate the RMH and MDACC prognostic scores with survival outcome in this population.

## RESULTS

### Patient Characteristics

A total of 40 patients were included in this retrospective review. The baseline characteristics of these patients, divided into categories for the univariate analysis, are summarized in Table 1. The median age at presentation was 15 years (range 2-17 years). Twenty-one patients (53%) were male. Only 3 patients (8%) had not received any systemic therapy for their disease (two of them received frontline surgical and/or radiation therapy)before being evaluated for phase I therapy; the patients' diagnosis were retroperitoneal alveolar sarcoma, nasopharyngeal papillar adenocarcinoma and melanoma of the scalp. Among the 37 patients who had received at least 1 prior treatment, the median number of prior treatments was 3 (range 1-7). Most patients (90%) had solid tumors. The most common cancer type was Ewing sarcoma (N=6, 15%) (Table 2).

**Table 1:** Univariate analysis of associations between baseline patient characteristics and overall survival (n = 40; median overall survival of whole group, 8.5 months)

Baseline characteristic	No. (%)	Median overall survival, months	P
Age			0.034
<15 years	19 (47)	5.6	
≥15 years	21 (53)	12.3	
Sex			0.780
Female	19 (47)	10.4	
Male	21 (53)	5.5	
Eastern Cooperative Oncology Group performance status			0.003
0-1	36 (90)		10.4
2	4 (10)		1.3
Hemoglobin			0.010
<10.5 g/dL	12 (30)	2.7	
≥10.5 g/dL	28 (70)	10.4	
Platelets			0.760
≤440 × 103/μL	26 (65)	8.5	
>440 × 103/μL	14 (35)	5.1	
Albumin			0.0058
<3.5 g/dL	8 (20)	2.4	
≥3.5 g/dL	32 (80)	10.6	
Number of prior therapies			0.300
0-3	27 (68)		10.4
>3	13 (32)		6.7
Prior radiation therapy			0.550
No	13 (32)	3.6	
Yes	27 (68)	10.4	
Number of metastatic sites			0.960
0-1	24 (60)		10.6
≥2	16 (40)		8.5
Lactate dehydrogenase			0.21
≤618 IU/L	26 (35)	10.4	
>618 IU/L	14 (65)	5.5	
Royal Marsden Hospital score			0.023
0 or 1	33 (82)	9.5	
>1	7 (18)	1.9	
MD Anderson Cancer Center score			0.350
0 or 1	20 (50)	8.5	
>1	20 (50)	6.2	

**Table 2:** Tumor types observed in the patient population (n = 40), according to histologic findings

Tumor type	No. (%)
Solid tumors (non CNS)	22 (55)
Ewing sarcoma	6 (15)
Desmoplastic small round cell tumor	3 (8)
Alveolar soft tissue sarcoma	2 (5)
Hepatocellular carcinoma	2 (5)
Osteosarcoma	2 (5)
Epitheloid sarcoma	1 (3)
Melanoma	1 (3)
Mixoid sarcoma	1 (3)
Nasopharyngeal carcinoma	1 (3)
Neuroblastoma	1 (3)
Papillary adenocarcinoma	1 (3)
Squamous cell carcinoma	1 (3)
Solid tumors (CNS)	14 (35)
Glioblastoma multiforme	5 (13)
Anaplastic ependymoma	3 (8)
Medulloblastoma	3 (8)
Diffuse pontine glioma	1 (3)
Meningioma	1 (3)
Primary neuroecatodermal tumor	1 (3)
Liquid tumors	4 (10)
Burkitt lymphoma	2 (5)
T-cell lymphoma	1 (3)
Hodgkin lymphoma	1 (3)

### Treatments

All patients received treatment in at least 1 phase I trial (range 1-5). Of 40 patients, 27 patients participated in 1 protocol, 9 in 2 protocols, 3 in 3 protocols, and 1 in 5 protocols. The agents used in each protocol are listed in Table S1. This table reflects the types of therapy the patients received. For instance, the same patient could have been on 5 different types of clinical trials when sequentially administered on progression from one trial to another. Of the 59 different protocol trials that were used, 56 (95%) were on trials that included at least 1 targeted agent and 3 (5%) included a cytotoxic agent only.

### Survival Analyses

**Figure 1:**
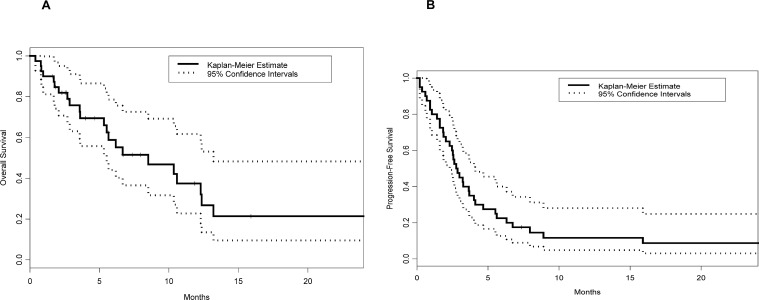
Survival of cancer patients age <18 treated on phase I clinical trials at MD Anderson Cancer Center 2005-2012 (n = 40) A) Overall survival B) Progression free-survival

Among 40 patients, 22 died. The median overall survival duration from the time of enrollment in a phase I trial was 8.5 months (95% confidence interval, 5.5-13.2 months; Figure 1A. Factors associated with decreased overall survival in univariate analysis were age younger than 15 years (P = 0.034), ECOG performance status ≥2 (P = 0.003), hemoglobin < 10.5 g/dL (P = 0.010), albumin < 3.5 g/dL (P = 0.0058), and RMH score >1 (P = 0.023; Table 1).

The final multivariate Cox proportional hazards model showed that age ≥15 years (P = 0.0065), was independently predictive of increased overall survival duration. More than 3 prior therapies (P = 0.053) was predictive of decreased overall survival duration; the fact that this variable did not reach the usual significant cutoff, might be related to the fact that our cohort was small. A larger population will be needed to adequately validate this variable(Table 3).

**Table 3:** Independent predictors of increased or decreased overall survival and progression-free survival according to multivariate analysis

	Harard ratio (95%	
Risk Factors	confidenceinterval)	P
Overall Survival		
Age ≥ 15 years	0.3 (0.1-1.1)	0.006
>3 prior therpies	2.6 (1.0-6.9)	0.053
Prior radiation therapy	0.4 (0.1-1.1)	0.083
Progression-free survival		
Age ≥ 15years	0.3 (0.1, 0.6)	<0.001
Performance Status >1	12 (3.4, 44)	<0.0001
>3 prior therapies	3.5 (1.6, 7.8)	0.002
Prior radiation therapy	0.4 (0.2, 0.99)	0.049

**Table 4:** Phase I treatments used in the patient population

Type of treatment	No. of pa tients (%)
Single-agent targeted therapy	6 (10.2)
Monoclonal antibody against EGFR	2 (3.4)
IGF-1R-targeted antibody	1 (1.7)
Chimeric monoclonal antibody targting CD30	1 (1.7)
Tyrosine kinase inhibitor	1 (1.7)
mTOR inhibitor	1 (1.7)
Targeted therapy combination	27 (45.7)
Angiogenesis inhibitor, PARP inhibitor	1 (1.7)
Angiogenesis inhibitor, mTOR inhibitor	8 (13.5)
Angiogenesis inhibitor, tyrosine kinase inhibitor	1 (1.7)
Angiogenesis inhibitor, tyrosine kinase inhibitor, mTOR inhibitor	1 (1.7)
Angiogenesis inhibitor, tyrosine kinase inhibitor, monoclonal antibody	1 (1.7)
Tyrosine kinase inhibitor, tyrosine kinase inhibitor	1 (1.7)
Tyrosine kinase inhibitor, histone deacetylase inhibitor	6 (10.1)
Tyrosine kinase inhibitor, histone deacetylase inhibitor, monoclonal antibody	1 (1.7)
Tyrosine kinase inhibitor, mTOR inhibitor	3 (5.1)
mTOR inhibitor, monoclonal antibody	4 (6.8)
Targeted therapy combined with chemotherapy	23 (39)
Angiogenesis inhibitor, bendamustine	1 (1.7)
Angiogenesis inhibitor, gemcitabine, protein-bound paclitaxel	1 (1.7)
Angiogenesis inhibitor, mTOR inhibitor, liposomal doxorubicin	2 (3.4)
Angiogenesis inhibitor, oxaliplatin, 5-fluorouracil	1 (1.7)
Hypomethylating agent, 5-fluorouracil	1 (1.7)
Hypomethylating agent, irinotecan	1 (1.7)
Hypomethylating agent, valproic acid	3 (5.1)
Proteosome inhibitor, liposomal doxorubicin, gemcitabine	9 (15.2)
Proteosome inhibitor, mTOR inhibitor, topotecan	2 (3.4)
Tyrosine kinase inhibitor, valproic acid	2 (3.4)
Chemotherapy only	3 (5.1)
Cisplatin, liposomal doxorubicin	1 (1.7)
Aerosolized IL-2	1 (1.7)
Temozolomide, pegylated interferon alpha	1 (1.7)

The median progression free-survival was 2.8 months (95% confidence interval, 2.3-4.1 months; Figure 1B. Factors associated with decreased progression-free survival in the univariate analysis were age younger than 15 years (P = 0.028), ECOG performance status ≥2 (P = 0.045), more than 3 prior therapies (P = 0.090), hemoglobin < 10.5 g/dL (P = 0.013), and albumin levels < 3.5 g/dL (P = 0.05). The multivariate analysis showed that age ≥15 years (P < 0.001) and prior radiation therapy (P = 0.049) were independently predictive of increased progression free-survival duration and ECOG performance status >1 (P < 0.001) and more than 3 prior therapies (P = 0.002; Table 3) were predictive of decreased progression free-survival duration.

### Prognostic Scores Validation

**Figure 2:**
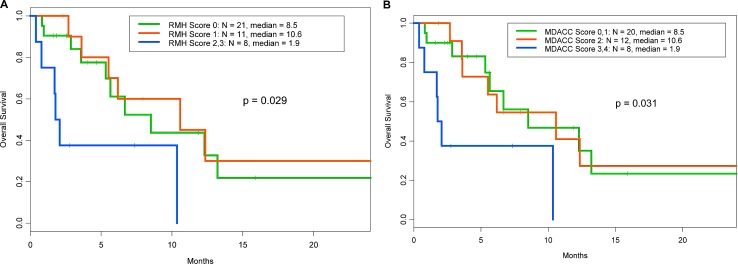
A) The Royal Marsden Hospital (RMH) risk stratification is based on LDH level albumin level, and number of metastatic sites. Assignment to groups 0 and 1 was associated with significantly better survival than groups 2 and 3 (P = 0.029).B) The MD Anderson Cancer Center (MDACC) score is based on LDH level, albumin level, ECOG performance status, number of metastatic sites and presence of gastrointestinal tumor. Patients with a score of ≥ 3 had decrease median survival rates than patients with a score <3.

To validate the RMH score in our pediatric population, we divided the patients based on the number of positive variables (LDH levels higher than the upper limit of normal (>618 IU/L), albumin <3.5 g/dL; and more than 2 metastatic sites of disease). 21 patients had a score of 0, 11 had 1, 7 had 2 and only 1 patient had a score of 3. Interestingly, RMH score ≥ 2 was associated to poor overall survival (median = 1.9 months; P = 0.029), there was not significant survival difference between patients with scores of 0 or 1 (8.5 and 10.6 months respectively); Figure 2A. For MDACC score validation, patients were assigned to 1 of 5 risk groups on the basis of their number of presenting risk factors: 0, low risk; 1, low-intermediate risk; 2, intermediate risk; 3, high-intermediate risk; and 4 or 5, high risk. 5 patients had a score of 0, 15 had a score of 1, 12 had a score of 2, 6 had a score of 3, 2 had a score of 4; none of the patients had a score of 5. The survival curves for these risk groups are shown in Figure 2B; for analysis, we collapsed groups 0 and 1 as well as groups 3 and 4 (since groups 0 and 4 had too few cases). The median survival for patients in groups 0-1 (20 patients) was 8.5 months, patients in group 2 (12 patients) had a median survival of 10.6 months and patients in groups 3-4 (8 patients) had a median survival of 1.9 months (P = 0.031).

### Molecular Profiling Results

Samples from 14 patients were submitted for molecular analyses (Table S2). Of the 5 patients tested for *PIK3CA* mutations, only 1 tested positive; this patient had squamous carcinoma of the lip, which progressed within 1 month after the targeted therapy was started. A mutation was detected in codon 542 of PIK3CA (GAA to AAA), which changed the encoded amino acid from Gly to Lys (E542K). Eight patients were tested for *BRAF* mutations in codons 595-600 of exon 15 of the *BRAF* oncogene; 1 patient tested positive for the BRAF V600E mutation (melanoma).

## DISCUSSION

Our primary objective for this analysis was to describe the clinical characteristics of pediatric patients who were enrolled in phase I trials and to determine whether pre-enrollment clinicopathologic characteristics had an impact on survival outcomes. Our results showed that both the RMH and MDACC scores can be used to measure survival outcomes in pediatric patients enrolled in investigational therapies. However, a better composite score using a larger dataset is warranted.

Pediatric patients enrolled in our phase I trials were heavily pretreated; 26 patients (65%) had received both prior radiation therapy and prior chemotherapy and 36 patients (90%) had previously received 2 or more prior chemotherapy regimens. Only 3 of the patients (8%) had received no prior therapy. These results are similar to those reported in a prior study, in which 68% of pediatric patients received both radiation therapy and chemotherapy before entering a phase I trial.[17] Despite this heavy pretreatment history, most of the patients had a good performance status (90% of patients had an ECOG performance status <2).

**Table 5:** Molecular analyses conducted for patients treated in phase I trials

Molecular mutation or aberration	No. patients tested	No. patients who tested positive
c-MYC	2	1
BRAF	8	1
PI3KCA	5	1
c-MET	2	2
EGFR	1	1

The number of prior therapies may also affect the response to molecularly targeted agents. Patients may do better when matched to a molecularly targeted therapy earlier in the course of their disease.[18] Recent data support the practice of treating adult patients with molecularly matched targeted agents based on molecular profile or pathway aberrations identified.[19-21] It will be important to determine whether such matching independently affects survival outcomes in pediatric patients in early phase clinical trials.

The median overall survival duration of 8.5 months (95% confidence interval, 5.5-13.2 months) that we observed in our patient population is longer than that reported in a prior multi-institutional report (i.e., about 5 months).[1] More importantly, the phase I therapy itself did not cause mortality.

There is a clear need to identify patients who are at risk of early death and thereby improve patient selection for phase I trials.[13, 14] The RMH score, a prognostic model for overall survival in adult patients treated in phase I trials, was proposed on the basis of a retrospective review of 212 patients treated in phase I studies. In this review, Arkenau et al found that elevated LDH levels, low albumin levels, and >2 sites of metastasis were independent prognostic factors for poor survival.[14] The RMH score suggests that patients with a score of 0-1 have significantly longer overall survival durations than patients with a score of 2-3. In our analysis, the RMH score was not identified as an independent prognostic factor for overall or progression-free survival in the multivariate analysis, although it was found to be a significant prognostic factor for overall survival in the univariate analysis (P = 0.029); however, the fact that the first two groups (score 0 and 1) had very similar survival curves indicates that the RMH score does not discriminate well between low and moderate risk patients. Our univariate analysis showed that low hemoglobin levels (<10.5 g/dL) also predicted decreased overall survival. This was not observed in the analysis performed by Arkenau et al; however, they used a higher cutoff for hemoglobin (12 g/dL) and the study mostly focused in adult population

The MDACC score was first reported in 2012 by Wheler et al.[13] They found that the addition of 2 prognostic factors, ECOG performance status ≥1 and presence of a gastrointestinal tumor, strengthened the RMH score. Similar to our RMH score results, our multivariate analysis showed that MDACC score was not an independent prognostic factor for overall survival in a pediatric population, but it was also found to be a significant prognostic factor for overall survival in the univariate analysis (P = 0.031); due to the fact that this 40-patient dataset does not have enough patients within the low risk (MDACC score 0) or high risk (MDACC score 4 or 5) categories; one important weakness of the study is the fact that gastrointestinal tumors (a variable on the MDACC scale) are not common in the pediatric population. A bigger dataset is warranted to adequately validate this prognostic score.

Our study represents a single institutional experience, which allowed for detailed analysis of patient information. However, a potential limitation of the study is the ECOG performance status. Performance status was subjectively scored by treating physicians at the time of patient enrollment in the phase I trial. The Lansky play scale or Karnofsky score are usually used in pediatrics; these scores are more validated than ECOG performance status for use in pediatric patients. Future studies should include Lansky play scale or Karnofsky score as a performance status score in addition to ECOG performance status. We also studied a very heterogeneous population in varied different types of clinical trials.

As this is a retrospective study, we found that a few patients were tested for mutations whenever they were referred to Phase I trials. This was limited based on archival sample availability. Unfortunately only few patients (n= 14) actually had genetic studies and only a few of them were actionable making difficult to correlate these results with outcomes. In addition most of the patients were older adolescents and the median PFS of more than 1 cycle which is close to adult phase 1 trials may reflect this. Interestingly the adult MDACC prognostic score includes gastrointestinal location tumors (a variable on the MDACC scale) are not common in the pediatric population. This is likely one of the reasons the MDACC score was not found to be significant as pediatric tumors infrequently involve the GI tract.

Progress in the treatment of pediatric cancer depends on the safe and efficient development of new therapeutic agents. Our findings indicate that although the parameters used in RMH and MDACC scores may help physicians select the best pediatric patients for phase I trials a novel pediatric prognostic score is needed. Use of these scores may also generate more complete and clear discussions between physicians and patients on the risks and benefits of enrolling in phase I trials. A prospective study to develop a composite score using the MDACC and RMH scores in the context of a consistent and larger group of pediatric patients and enrolled in similar clinical trials is warranted. In the era of targeted therapy more trials should allow pediatric patients earlier in the drug development especially if deemed safe in adults in early phase trials.

## METHODS

### Patient Characteristics

This study was performed in accordance with the guidelines of The MD Anderson Cancer Center Institutional Review Board (IRB). Since this was a retrospective study, IRB waived the need for informed consent. We reviewed the clinical outcomes of 40 patients younger than 18 years who were treated in the Division of Pediatrics or the Department of Investigational Cancer Therapeutics as part of the MD Anderson Cancer Center Phase I Clinical Trials Program between January 2005 and January 2013. Patient records were reviewed for medical history, laboratory findings, and clinical findings at the time of initial presentation for an adult focused phase I trial (i.e., at baseline), as well as for treatment(s) given and clinical outcomes (i.e., overall survival and progression-free survival).

Baseline characteristics collected included age, sex, tumor type, ECOG performance status, number of prior systemic therapies, number of metastatic sites, hemoglobin levels (g/dL), LDH levels (IU/L), and albumin levels (g/ dL); platelet count (103/μL) and number of prior radiation treatments, date of initiation of phase I therapy, date of relapse, and date of death or last follow-up were also recorded.

To determine the RMH prognostic score for each patient, we added points according to the findings of 3 variables: normal LDH levels (0) or LDH levels higher than the upper limit of normal (>618 IU/L), (+1); albumin ≥ 3.5 g/dL (0) or albumin <3.5 g/dL (+1); and 2 or fewer metastatic sites of disease (0) or more than 2 metastatic sites of disease (+1). To determine the MDACC prognostic score for each patient, we added points according to the findings of 5 variables: LDH ≤ 618 IU/L (0) or LDH > 618 IU/L (+1); albumin ≥ 3.5 g/dL (0) or albumin < 3.5 g/ dL (+1); 2 or fewer metastatic sites of disease (0) or more than 2 metastatic sites of disease (+1); ECOG performance status = 0 (0) or ECOG performance status ≥1 (+1); and no gastrointestinal tumor (0) or gastrointestinal tumor present (+1).

### Molecular Analysis

With the evolution of clinical molecular profiling, molecular aberrations for hot-spot mutations in specific genes—*BRAF, PIK3CA, c-MET* and *EGFR*—were investigated using available archival formalin-fixed, paraffin-embedded tissue blocks or material from fine-needle aspiration biopsy obtained from diagnostic or therapeutic procedures. All histologic findings were centrally reviewed at MD Anderson. Mutation testing was done in the Clinical Laboratory Improvement Amendment (CLIA)–certified Molecular Diagnostic Laboratory within the Division of Pathology and Laboratory Medicine at MD Anderson. *n-MYC* (MYCN) amplification was performed on specimens from patients with neuroblastoma according to established CLIA-certified procedures in the MD Anderson pathology laboratory. DNA was extracted from microdissected paraffin-embedded tumor tissue and analyzed using polymerase chain reaction and a pyrosequencing method for analyses of the specific oncogenes. The sensitivity of detection of this assay was approximately 1 in 10 mutation-bearing cells in the microdissected area.

### Statistical Analysis

Patient characteristics were summarized using medians and ranges for continuous variables and frequencies and percentages for categorical variables. The median progression-free (PFS) and overall survival (OS) durations were estimated using the Kaplan-Meier method. Overall survival was calculated from the date a patient started therapy in the phase I program to the date of death; patients who were alive at the time of this analysis were censored on that date. Progression-free survival was calculated from the date a patient started phase I therapy to the date of documented relapse or death; patients who were alive and relapse-free at the time of this analysis were censored on that date. Univariate and multivariate Cox proportional hazards models were fit to assess associations between patient characteristics and clinical outcomes (i.e., overall and progression-free survival). In the multivariate analysis, a backward variable selection procedure was conducted to identify the optimal set of independent variables for OS and PFS. P values ≤ 0.05 were considered significant for all statistical analyses. The analyses were performed by K.H. using Spotfire S+8.2 for Windows software (TIBCO Software Inc.)
